# Experiment dataset of dynamic properties of damper material derived from automotive spare parts as passive control devices for retrofitting existing buildings

**DOI:** 10.1016/j.dib.2023.109166

**Published:** 2023-04-20

**Authors:** Yenny Nurchasanah, Bambang Suhendro, Iman Satyarno

**Affiliations:** a Department of Civil and Environmental Engineering, Universitas Gadjah Mada, Yogyakarta, Indonesia; b Department of Civil Engineering, Engineering Faculty, Universitas Muhammadiyah Surakarta, Kartasura 57102, Indonesia

**Keywords:** Shock absorber, Engine mounting rubber, Rubber characteristic, Free vibration analysis, Stiffness, Damping ratio Structural dynamic

## Abstract

The data collection provides two clusters: rubber materials and dampers as passive energy dissipation devices that are selected from existing systems in automotive spare parts, namely rubber from engine mounting rubber, shock absorbers, and engine mounting rubber (EMR). Variable depending on the brand, number, and size of the rubber used by the manufacturer. Data on EMR rubber materials, such as ultimate tensile strength, axial elongation, hardness, and density. Data for EMR as a system, including data from static and dynamic tests. The parameters measured are stiffness and damping ratio. The area and shape of the hysteresis curve are used to determine the damping ratio. The data presented in the article will allow researchers to validate the dynamic models for several designs of dampers, such as a damper with a single EMR and a damper with a group of EMR systems.

**Specifications Table**

**Table:** 

Subject	Civil and Structural Engineering
Specific subject area	Damper, Shock absorber, Engine mounting rubber, Free vibration analysis, Structural Dynamic
Type of data	Table Graph Figure
How the data were acquired	Static and dynamic data of engine mounting rubber damper were acquired with accelerometer, load cell, and LVDT. The measured signals are calibrated with The Dewesoft data acquisition system with Dewesoft X3 software.
Data format	Raw, Analyzed
Description of data collection	Measure the tensile strength, break elongation, hardness, and density of the rubber of EMR to characterize the properties. Measure the stiffness and damping ratio parameters of EMR damper using load-displacement relationship data and the hysteresis curve area.
Data source location	Data collected from the Structural Laboratory of Department of Civil and Environmental Engineering Universitas Gadjah Mada in Yogyakarta Indonesia, the PT Kayaba Astra Indonesia Laboratory, and the Center for Leather, Rubber, and Plastics Laboratory in Yogyakarta Indonesia.
Data accessibility	Data are provided in this article. Direct URL: https://doi.org/10.17632/f78s4xcbyg.1 DOI: 10.17632/f78s4xcbyg.1

## Value of the Data

• Data can be used to inform researchers and building construction professionals about the importance of seismic mitigation and rehabilitation due to the high risk of earthquakes, especially in developing countries that can get damper devices easily and cheaply.
• Data can be used to guide the designers and construction industries on the concepts of retrofitting existing buildings with damper devices, which is very necessary in seismic design.
• Researchers that work with modeling software and computational procedures can use the data to improve the performance of existing buildings and remodel them, ensuring that the structures are designed to withstand earthquakes.
• Data can be employed in choosing the most appropriate damper devices to be considered in retrofitting low-rise buildings or simple houses, with the advantage of ease of installation and no expertise required.

## 1 Objective

Data is collected for the purpose of enabling engineers and researchers to develop simple earthquake dampers.

## 2 Data Description

### 2.1 Shock Absorber (SA) Data Test

**Fig. 1:**
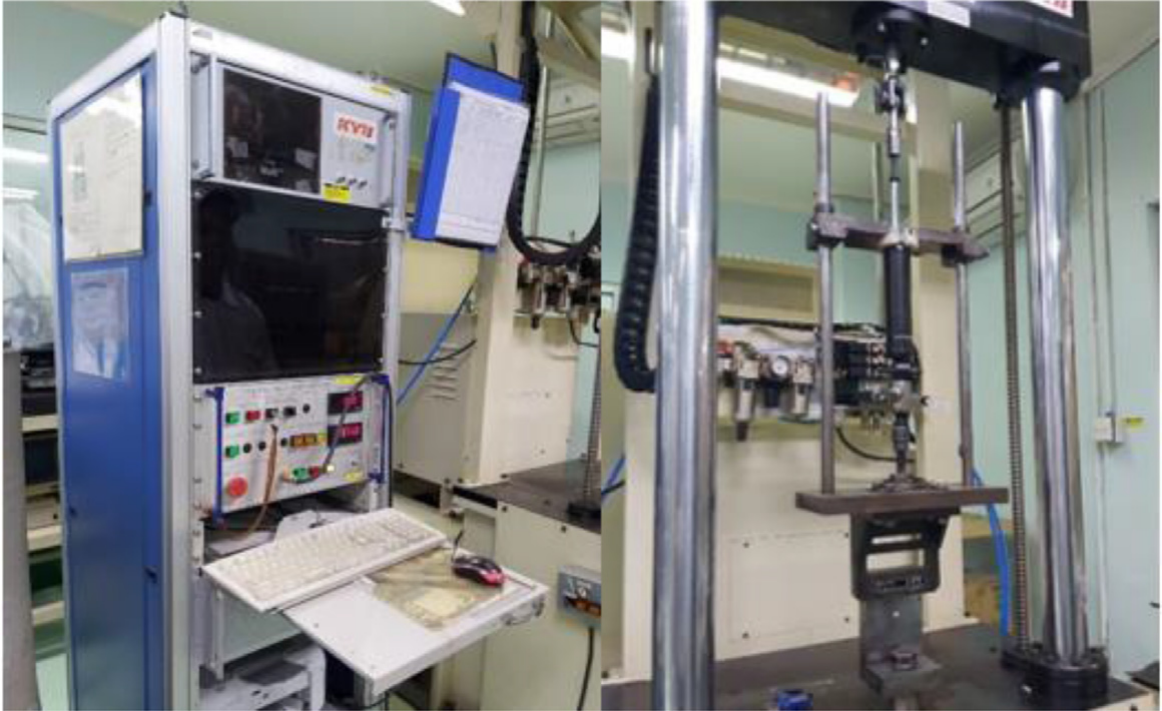
Setting the shock absorber specimen on the Auto Damping Force Tester machine.

Table 1, Table 2, Table 3, Table 4, Table 5 illustrates the specimens of Shock absorbers variant (see Table 11) that were tested using an auto-damping force tester (see Fig. 1) with certain displacement controls and velocity speeds from 0.05 m/s to 0.6 m/s. The parameters observed are the rebound and compress values, which are the resistance values of the fluid system due to being given a pulling and pushing force at a certain speed (7 cycles).

**Table 1:** Shock Absorbers (MG 14) KYB Astra, Hino Dutro Truck (rear - Gross Vehicle Weight 8250kg).

Variant	SA - MG 14 - KYB Astra, Hino Dutro Truck (rear - Gross Vehicle Weight 8250kg)								
n	1			2			3		
Span	±25mm			±25mm			±25mm		
cycle	Velocity	Force (N)		Velocity	Force (N)		Velocity	Force (N)	
	(m/s)	Rebound	Compress	(m/s)	Rebound	Compress	(m/s)	Rebound	Compress
1	0.05	363	137	0.05	330	135	0.05	337	128
2	0.10	917	335	0.10	807	301	0.10	776	283
3	0.20	1558	621	0.20	1433	619	0.20	1399	587
4	0.30	1760	732	0.30	1698	718	0.30	1678	693
5	0.40	1954	806	0.40	1868	791	0.40	1873	775
6	0.50	2092	889	0.50	2040	868	0.50	2049	845
7	0.60	2218	956	0.60	2200	934	0.60	2204	910

**Table 2:** RE 20 – SA Front Mitsubishi Kuda Car, Original Part Number MT 141246.

Variant	RE 20 – SA Front Mitsubishi Kuda Car, Original Part Number MT 141246								
n	1			2			3		
Span	±25mm			±25mm			±25mm		
cycle	Velocity	Force (N)		Velocity	Force (N)		Velocity	Force (N)	
	(m/s)	Rebound	Compress	(m/s)	Rebound	Compress	(m/s)	Rebound	Compress
1	0.05	1282	532	0.05	1145	543	0.05	1030	438
2	0.10	1931	901	0.10	1756	742	0.10	1630	632
3	0.20	2791	1415	0.20	2689	1246	0.20	2561	1083
4	0.30	3339	1693	0.30	3284	1572	0.30	3181	1468
5	0.40	3727	1939	0.40	3640	1837	0.40	3590	1753
6	0.50	4031	2130	0.50	3969	2032	0.50	3877	1951
7	0.60	4324	2281	0.60	4253	2189	0.60	4190	2109

**Table 3:** DJ and DJ-sp. SA Original - 280mm silver chrome Yamaha Jupiter motorcycle.

Variant	DJ dan DJ-sp. SA Original - 280mm silver chrome Yamaha Jupiter motorcycle								
n	1			2			3		
Span	±25mm			±25mm			±25mm		
cycle	Velocity	Force (N)		Velocity	Force (N)		Velocity	Force (N)	
	(m/s)	Rebound	Compress	(m/s)	Rebound	Compress	(m/s)	Rebound	Compress
1	0.05	131	33	0.05	59	43	0.05	44	46
2	0.10	268	35	0.10	162	42	0.10	139	43
3	0.20	610	36	0.20	470	42	0.20	405	40
4	0.30	1012	38	0.30	836	43	0.30	731	42
5	0.40	1582	43	0.40	1227	48	0.40	1079	44
6	0.50	1845	50	0.50	1594	53	0.50	1424	49
7	0.60	2230	60	0.60	1901	59	0.60	1712	57

**Table 4:** DJ and DJ-sp - SA Original without Spring - 280mm silver chrome Yamaha Jupiter motorcycle.

Variant	DJ dan DJ-sp - SA Original without Spring - 280mm silver chrome Yamaha Jupiter motorcycle								
n	1			2			3		
Span	±15mm			±15mm			±15mm		
cycle	Velocity	Force (N)		Velocity	Force (N)		Velocity	Force (N)	
	(m/s)	Rebound	Compress	(m/s)	Rebound	Compress	(m/s)	Rebound	Compress
1	0.05	125	154	0.05	124	123	0.05	124	103
2	0.10	151	157	0.10	140	125	0.10	141	105
3	0.20	417	168	0.20	390	134	0.20	378	114
4	0.30	857	183	0.30	700	146	0.30	636	123
5	0.40	1109	199	0.40	1042	158	0.40	999	140
6	0.50	1539	210	0.50	1398	167	0.50	1495	157
7	0.60	1948	229	0.60	1707	187	0.60	1692	187

**Table 5:** SA Yamaha Jupiter motorcycle (unoriginal).

Variant	SA Yamaha Jupiter motorcycle (unoriginal)								
n	1			2			3		
Span	±25mm			±25mm			±25mm		
cycle	Velocity	Force (N)		Velocity	Force (N)		Velocity	Force (N)	
	(m/s)	Rebound	Compress	(m/s)	Rebound	Compress	(m/s)	Rebound	Compress
1	0.05	14	11	0.05	11	16	0.05	9	12
2	0.10	28	15	0.10	23	17	0.10	20	15
3	0.20	129	20	0.20	107	23	0.20	91	18
4	0.30	200	29	0.30	160	28	0.30	145	22
5	0.40	264	36	0.40	223	33	0.40	177	31
6	0.50	296	41	0.50	306	39	0.50	245	37
7	0.60	404	47	0.60	339	43	0.60	237	42

### 2.2 Rubber Characterization Test

Table 6 illustrates two brands of EMR, HINO (V-H) and Mitsubishi (V-M) with their performance index of Tensile strength, Break elongation, Hardness, and Density. Tensile strength and break elongation specimens shaped as dumbbell (narrow section's length 20 mm and thickness 2 mm). The maximum stress at which the specimen will break is measured as force per unit of cross-sectional area. The greatest extension before the sample is broken is measured as the elongation at break (%), which is expressed as a percentage of the initial length.

**Table 6:** Engine mounting rubber characterization Test, V-H and V-M brand [1].

Performance Index	Test Value	
	V-H	V-M
Tensile strength (N/mm^2^)	17.53	16.79
Break elongation (%)	358.33	480
Hardness (shore A)	64.23	55.01
Density (gr.cm^3^)	1.19	1.14

### 2.3 Engine Mounting Rubber (EMR) Damper Static Test

Table 7 illustrates the stiffness values of various EMR rubber variants and their damper designs, consisting of nine EMR variants with 17 damper designs. Several designs were carried out by comparing the stiffness values collected in different laboratories.

**Table 7:** Engine mounting rubber damper stiffness.

EMR Damper Variant	Stiffness, k (N/mm)	
	Kayaba Astra Lab.	Structure Lab. of UGM
V-BP	392.62	-
V3-S	51.351	64.016
V3-4R	-	154.225
V4-S	70.393	103.351
V4-4R	-	401.342
V6-S	210.35	189.872
V6-4R	-	533.737
V7-S	39.665	19.008
V8-S	-	50.643
V8-2R	-	96.822
V8-4R	-	174.33
V9-S	-	99.082
V9-2R	-	187.05
V10-S	-	146.32
V10-2R	-	231.03
V11-S	-	86.802
V11-2R	-	149.35

### 2.4 Engine Mounting Rubber (EMR) Damper Dynamic Test

Table 8 is an analysis of the data from the free vibration test on seven EMR rubber variants with fifteen rubber number design variations (single -S, double -2R, and four rubber -4R). The damping ratio results (ξ, %) are taken from the decay behavior analysis.

**Table 8:** Damping ratio of free vibration test.

Varian EMR	ξ, %		
	One rubber (-S)	Two rubbers (-2R)	Four rubbers (-4R)
V3	11.901	**-**	17.293
V4	11.443	**-**	7.236
V6	9.295	-	8.801
V8	11.015	11.522	9.469
V9	11.141	12.841	-
V10	9.303	11.032	-
V11	14.986	13.666	-

Table 9 describes the data analysis of the damping ratio value to the stiffness value on the V6-S damper specimen with a displacement load input of 12.5 mm. performed on frequency cycles of 0.5, 1.0, 1.5, and 2.0 Hz.

**Table 9:** Damping ratio and stiffness of V6-S damper - displacement 12.5 mm.

Frequency, Hz	ξ, %	Stiffness, (N/mm)
0.5	6.923	206.75
1.0	8.478	211.23
1.5	8.906	201.85
2.0	9.237	201.91

Table 10 describes the data analysis of the damping ratio value to the stiffness value on the V6-S damper specimen with a displacement load input of 18.75 mm. performed on frequency cycles of 0.5, 1.0, 1.5, and 2.0 Hz.

**Table 10:** Damping ratio and stiffness of V6-S damper - displacement 18.75 mm.

Frequency, Hz	ξ, %	Stiffness, (N/mm)
0.5	6.544	204.9
1.0	6.033	210.02
1.5	6.382	204.07
2.0	5.950	204.65

## 3 Experimental Design, Materials and Methods

### 3.1 Shock Absorber Test

The shock absorber variant in Table 11 was carried out with a damping test using the Auto Damping Force Tester machine at PT Kayaba Astra Indonesia (Fig. 1). One variant is given a displacement control of 15 mm and the other 4 variants are given a displacement control of 25mm, all variants are given the same temperature conditions of 20°C. Temperature conditions must be measured because temperature greatly affects the fluid in the damper device, so that each specimen must be at the same temperature so that the results can be compared. In one testing stage, each specimen is given a velocity force with a speed level that changes from low to higher with a speed range between 0.05 m/s to 0.6 m/s.

**Table 11:** Shock absorber variant.

Varian *Shock Absorber*	Desain
Shock Absorber (MG 14, rear) KYB Astra, Hino Dutro Truck. Gross Vehicle Weight 8250 kg	
Shock Absorber (fore) Mitsubishi Kuda Car, Original Part Number MT 141246	
Shock Absorber - DJ dan DJ-sp (Original), 280mm silver chrome Yamaha (Jupiter) motorcycle	
Shock Absorber - DJ dan DJ-sp (Original), without Spring - 280mm silver chrome Yamaha (Jupiter) motorcycle	
Shock Absorber. Yamaha (Jupiter) motorcycle (unoriginal)	

### 3.2 Rubber Characterization Test

**Fig. 2:**
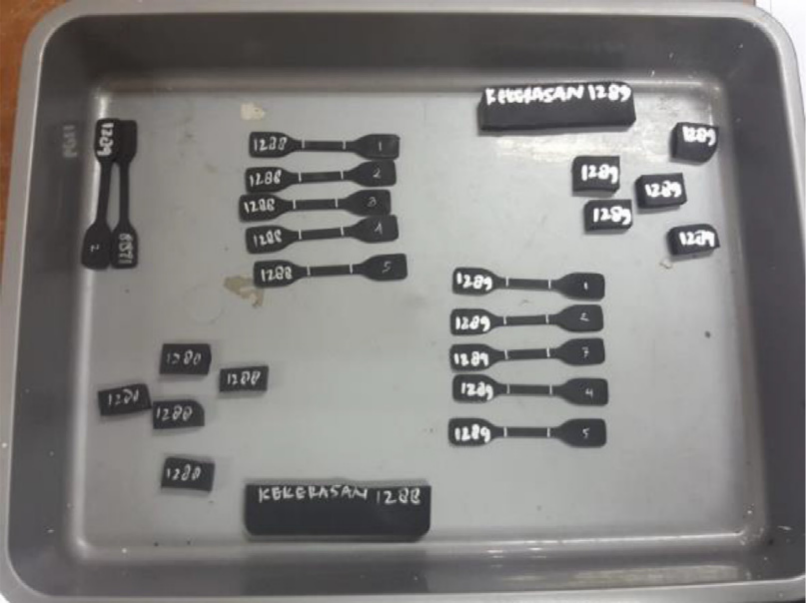
Rubber specimens of characterization test.

**Fig. 3:**
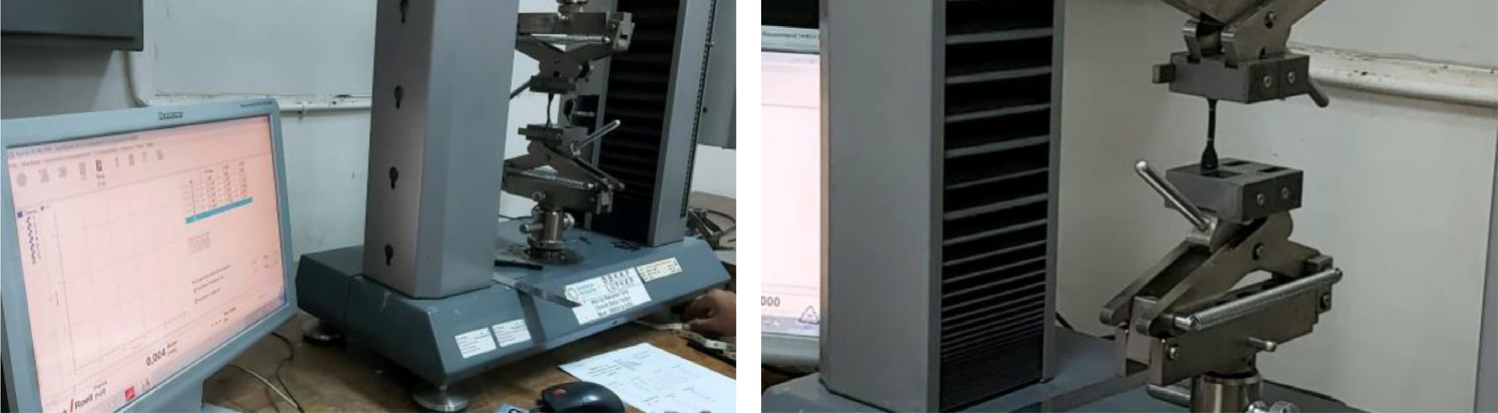
Tensile strength machine and elongation break.

**Fig. 4:**
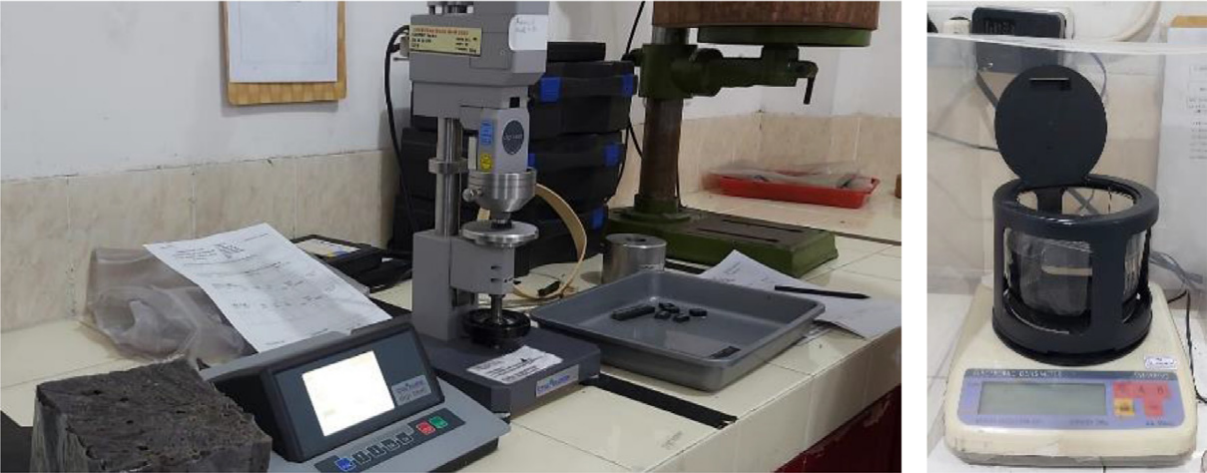
The automatic durometer hardness and Electronic Densimeter.

The experiment was carried out in the testing laboratory of Center for Leather, Rubber, and Plastics Yogyakarta, Indonesia. The test was carried out on two types of EMR rubber from two types of manufacturers with different brands of automotive spare parts manufacturers, brand from automotive companies HINO and Mitsubishi, namely V-H and V-M (Fig. 2). The tensile strength and break elongation test method in accordance with ISO 37: 2015 (IDT-2011) (Rubber, vulcanized or thermoplastic - determination of tensile stress-strain properties) [2] (Fig. 3). The hardness according to ISO 7619-1: 2010 (Rubber, vulcanized or thermoplastic Standard - measurement of identifying hardness - durometer method/Shore Hardness) [3] (Fig. 4). The density test uses an electronic densimeter with a resolution of 0.01 and follows ISO 2781: 2008 (Rubber, Vulcanized or Thermoplastic - Determination of Density) [4] (Fig. 4).

## 4 Engine Mounting Rubber Damper Test

### 4.1 First Stage (Static Test)

**Fig. 5:**
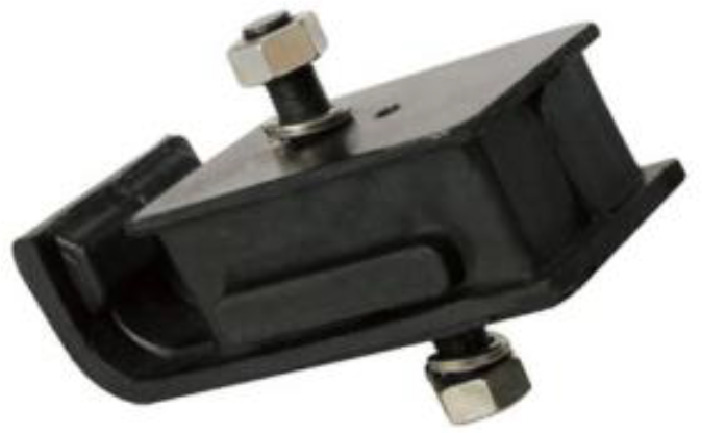
Engine Mounting Rubber (EMR).

**Fig. 6:**
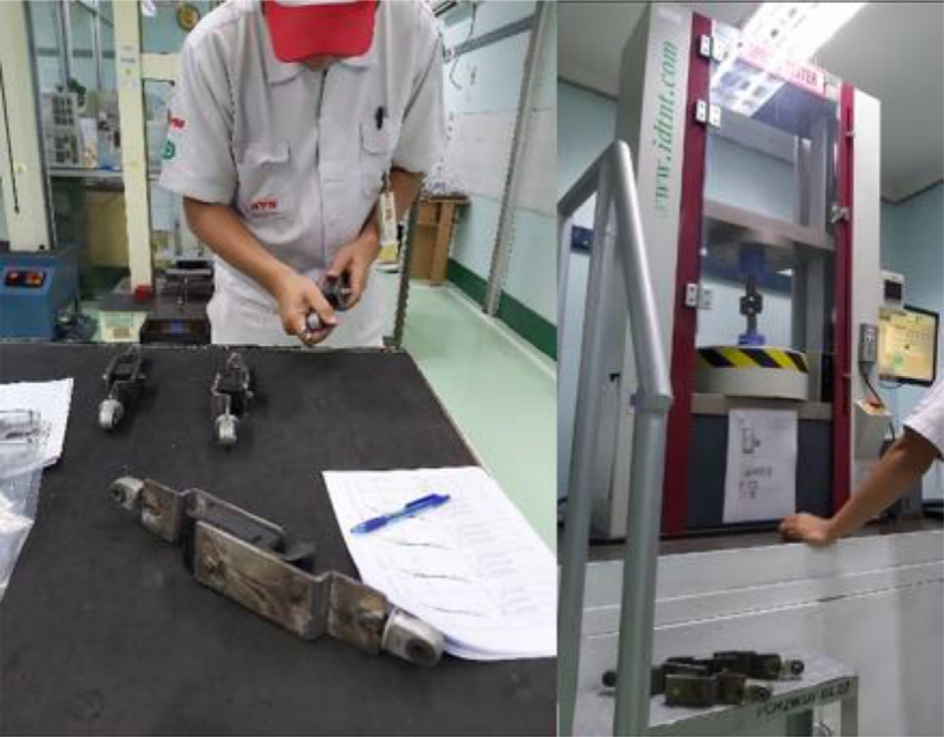
Setting the EMR damper specimen on the Spring Tester Machine.

Engine rubber mounting (EMR) (Fig. 5) is one of the vehicle components that is composed of high-damping rubber material that functions as a holder and damper for engine vibration so that vibration does not spread to the car frame, which then spreads to the passenger cabin. Experiments carried out at PT Kayaba Astra Bekasi Indonesia. Placing instrument as illustrated in Fig. 6. One of the holders (the upper side) is pulled in line with the damper device by controlling a specific displacement as required. The damper device is attached to both sides of the holder. By pulling the damper device, it will be possible to observe how the material responds to the pulling force and how significantly it has lengthened. Load values and displacement values are measured parameters that are digitally read by a computer. A tensile profile—a curve illustrating the relationship between the pulling force and the change in length—is obtained by pulling the material. The first stage of testing the damper properties was carried out on five variants of damper devices with rubber material (Table 12).

**Table 12:** Engine mounting rubber damper variant.

Damper Variant	Damper Design
V-BP elastomer bearing pad – modification	
V3 Viar engine mounting rubber (small)	
V4 Viar engine mounting rubber (medium)	
V6 engine mounting rubber colt diesel Canter Truck 4D32 Mitsubishi ME011832	
V7 rubber mounting rubber electric, engine pad – diesel engine mounting genset	

### 4.2 Second Stage (Static Test)

**Fig. 7:**
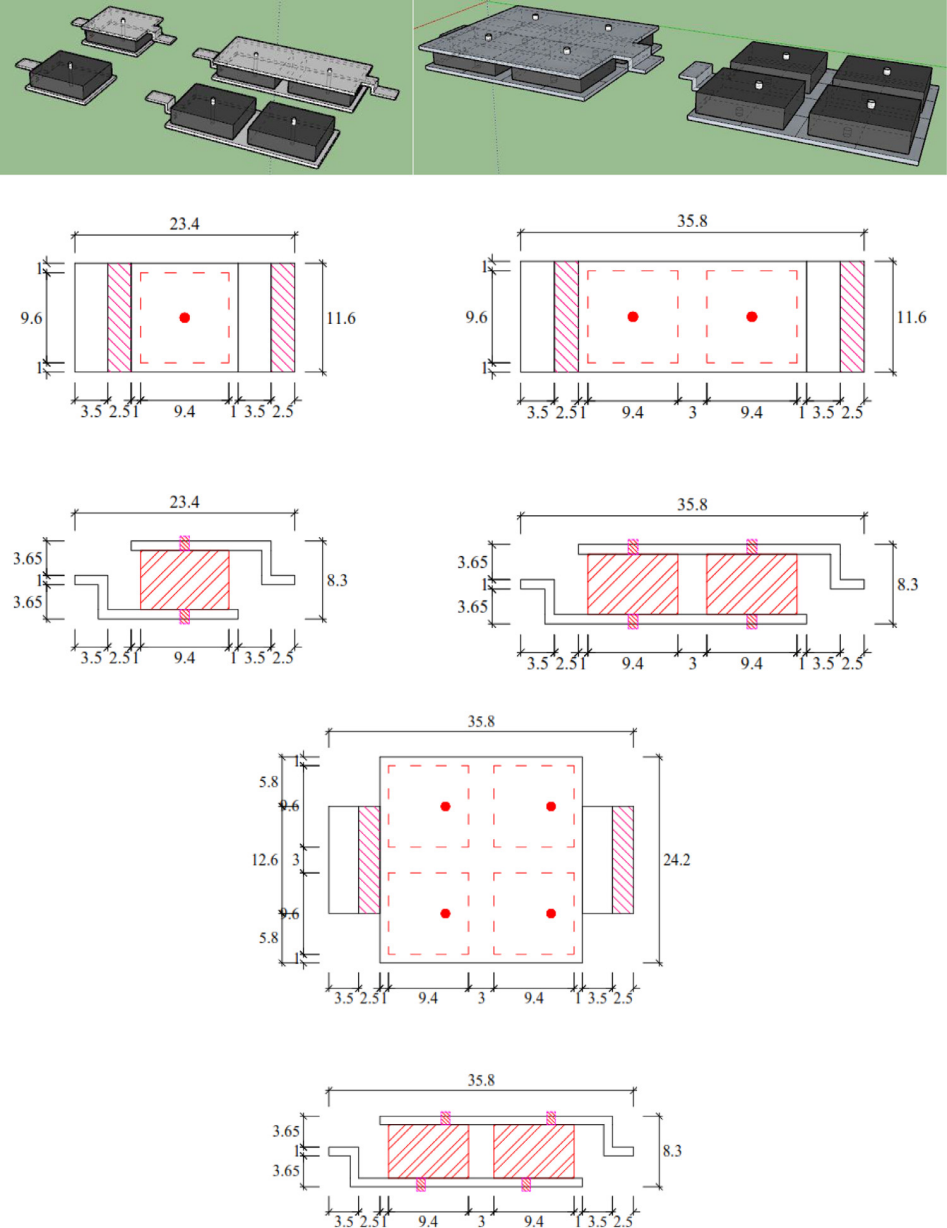
Damper Design Variant (one, two, and four EMR).

**Fig. 8:**
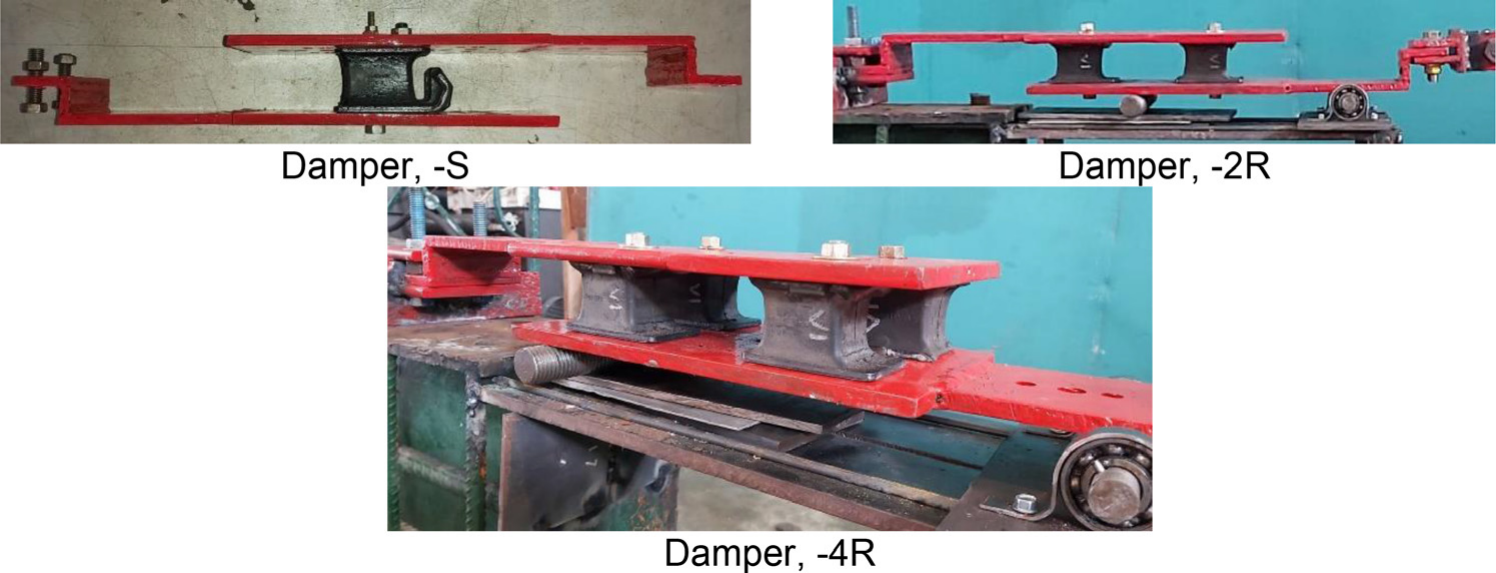
EMR Damper specimens (one, two, and four EMR).

**Fig. 9:**
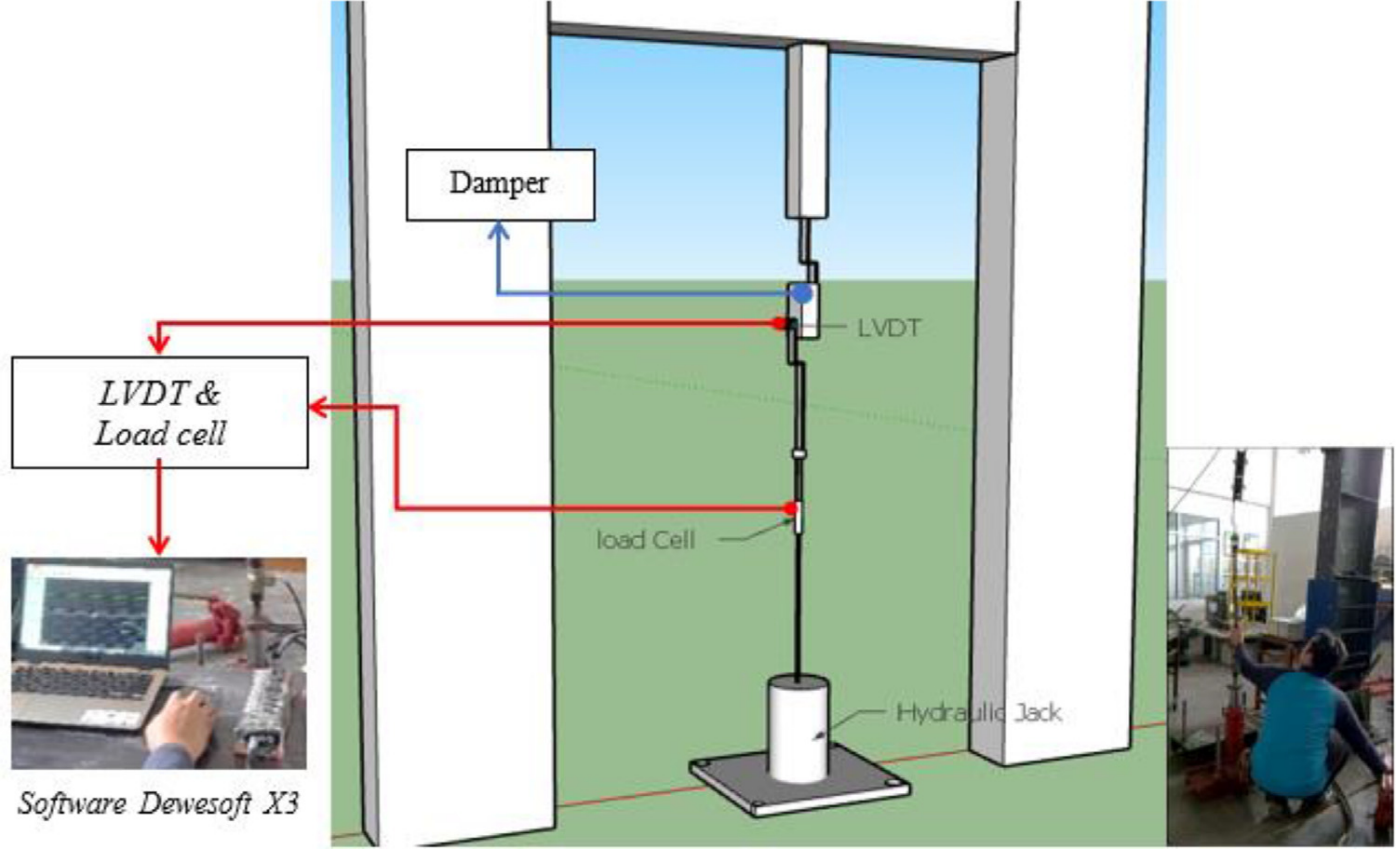
Scheme and setting of the EMR damper specimen on static test.

Carried out completely at the Gadjah Mada Structure Laboratory, setting up as shown in Fig. 9. The eighth of EMR variants and sixteenth of damper designs were tested (Table 12-13-14). The damper design was developed from one rubber, two rubbers, and four rubbers (Fig. 7-8). Static tests are carried out by using the system and instrumentation on a loading frame with the loading and unloading method. Two LDVTs are installed above and below the damper device to control and read displacement, respectively. Loading and unloading data is assisted by a load cell measuring instrument. Giving load and unload by pulling manually with a hydraulic jack. All LVDT measuring instruments and load cells are computerized using Dewesoft X3 software so that data on load values and displacement values can be obtained (Fig. 9).

**Table 13:** Engine mounting rubber variant.

V8	V9	V10	V11
HINO DUTRO 130	Hino 500 FG 215 JP (rear)	Hino 500 FG 215 JP (fore)	Truck Colt Diesel Mitsubishi canter double 125 PS

**Table 14:** EMR variant and damper design of static test.

EMR Varian	Damper Design		
	One rubber (-S)	Two rubbers (-2R)	Four rubbers (-4R)
V3	**√**	**-**	**√**
V4	**√**	**-**	**√**
V6	**√**	**-**	**√**
V7	**√**	**-**	**-**
V8	**√**	**√**	**√**
V9	**√**	**√**	**-**
V10	**√**	**√**	**-**
V11	**√**	**√**	**-**

### 4.3 Third Stage (Dynamic Test)

**Fig. 10:**
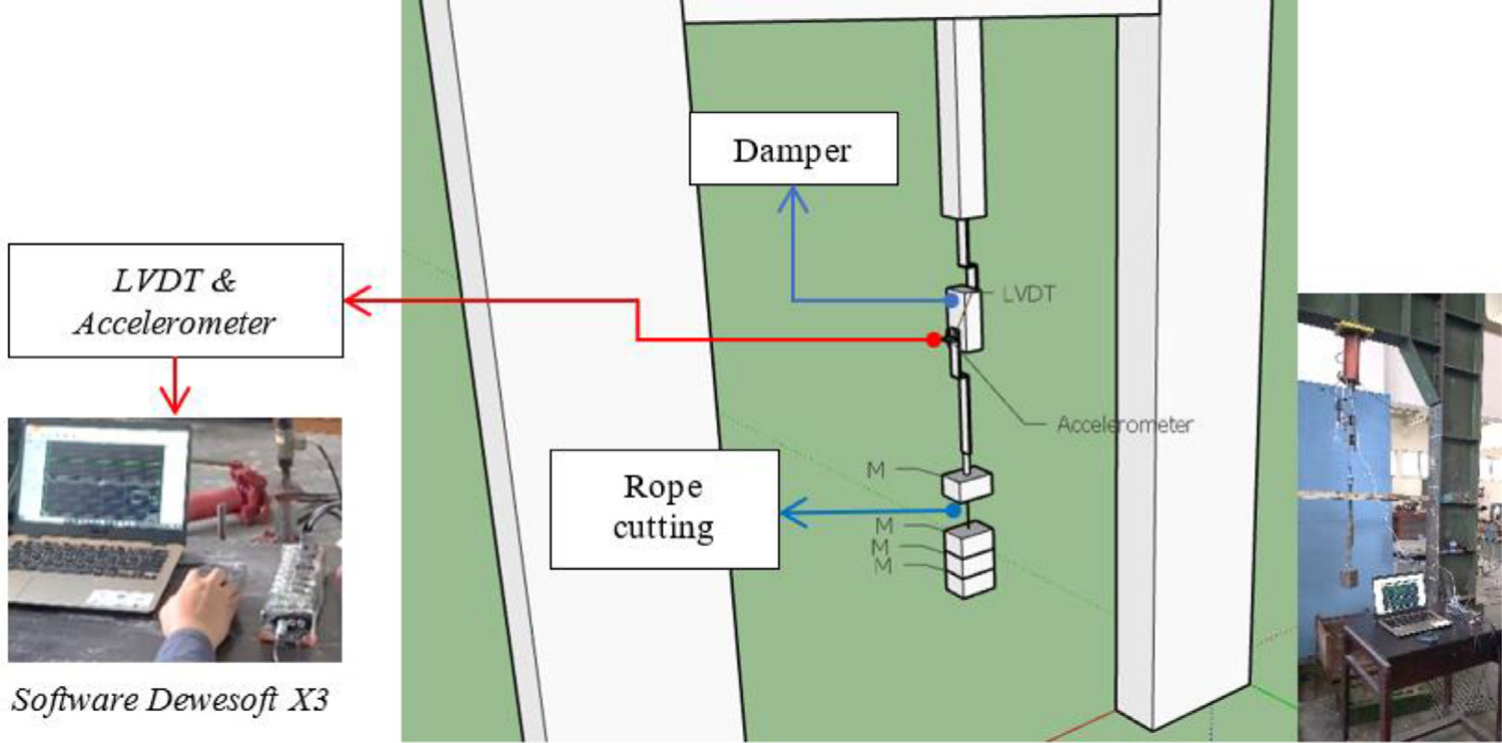
Scheme and setting of the EMR damper specimen on dynamic test.

**Fig. 11:**
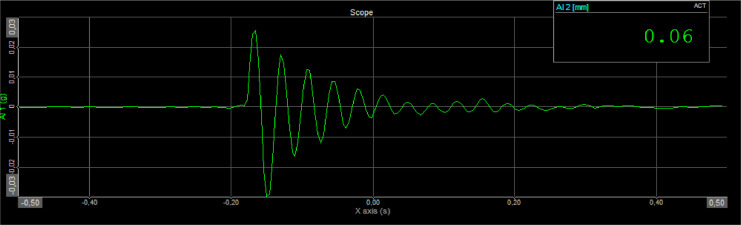
Time response to acceleration and frequency response to the free vibration test.

The dynamic test was carried out using two laboratory testing methods: the free vibration test and the shaking table test. A free vibration test was carried out to obtain decay behavior on the damper. The seven of EMR variants and fifteenth of damper designs were tested (Table 15). As seen on Fig. 10, The specimen is hung on the loading frame with one of the upper damper handles clamped and the other side of the bottom handle free. The load will be hung on the free side of the handle, with a rope serving as the connecting medium between the handle and the load. The rope will be cut to get a free vibration response from the damper. The accelerometer is placed on the side of the damper that is not clamped to obtain an acceleration response over time (Fig. 11). The decay behavior is obtained due to the material's role as a damper, and the frequency response is obtained by the Fast Fourier Transform (FFT).

**Fig. 12:**
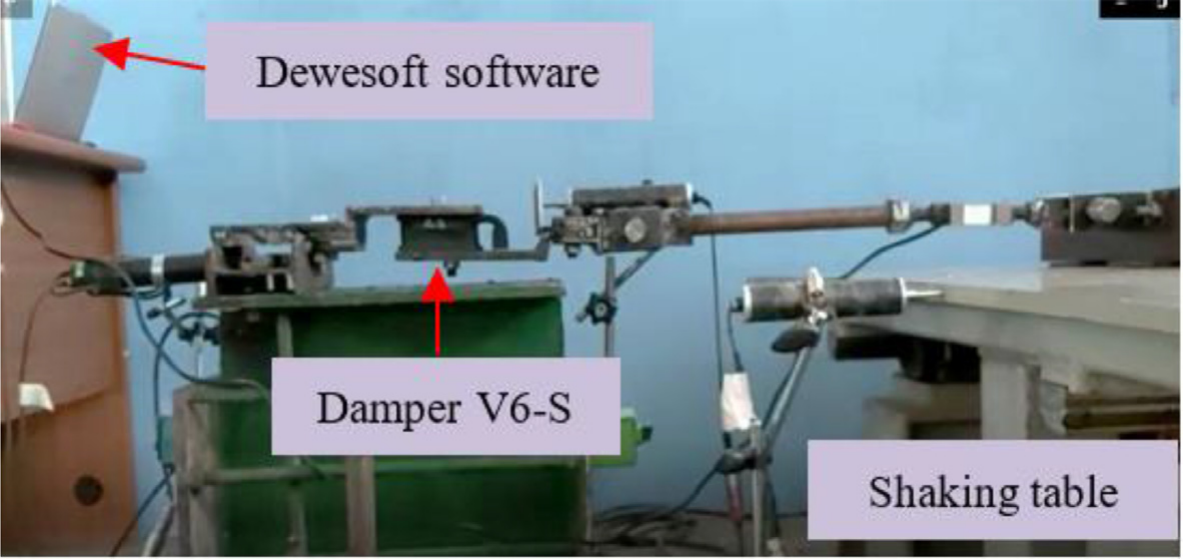
Dynamic test setting of EMR damper on a shaking table.

**Fig. 13:**
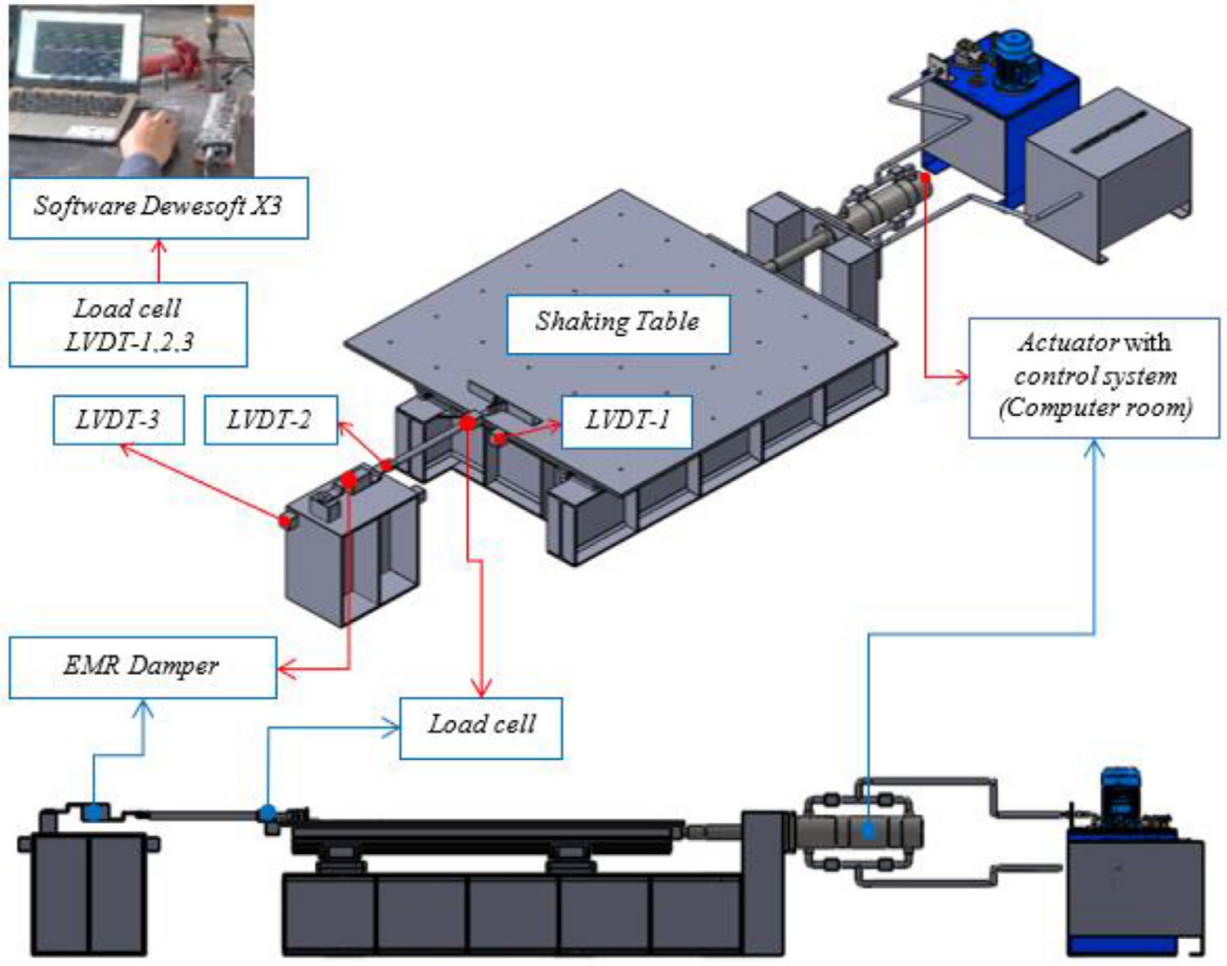
Scheme of dynamic test on a shaking table.

The dynamic test also involved the EMR damper material on a shaking table instrument with dancing earthquake software and Dewesoft X3 software (Fig. 12, Fig. 13). The load protocol on the shaking table input had two variations of displacement control (12.5 mm and 18.75 mm) and four variations of frequency (0.5 Hz, 1.0 Hz, 1.5 Hz, and 2.0 Hz). The damping ratio (ξ %) is calculated by calculating the area of the hysteresis loop curve.

**Table 15:** EMR variants and damper design of dynamic test.

EMR Varian	Damper Design		
	One rubber (-S)	Two rubbers (-2R)	Four rubbers (-4R)
V3	**√**	**-**	**√**
V4	**√**	**-**	**√**
V6	**√**	**-**	**√**
V8	**√**	**√**	**√**
V9	**√**	**√**	**-**
V10	**√**	**√**	**-**
V11	**√**	**√**	**-**

## Ethics Statements

The author has complied with Elsevier's 'Ethics in publishing' policy. Does not involve human subjects, animal experiments, and data collected from social media platforms.

## CRediT authorship contribution statement

**Yenny Nurchasanah:** Conceptualization, Methodology, Investigation, Writing – original draft, Writing – review & editing. **Bambang Suhendro:** Supervision, Conceptualization, Software, Writing – review & editing. **Iman Satyarno:** Supervision, Conceptualization, Software, Validation, Data curation, Writing – review & editing.

## Declaration of Competing Interest

The authors declare that they have no known competing financial interests or personal relationships that could have appeared to influence the work reported in this paper.

## Data Availability

Properties of Engine Mounting Rubber as Passive Control Device (Original data) (Mendeley Data).
